# How far musicality and perfect pitch are derived from genetic factors?

**DOI:** 10.1007/s13353-020-00563-7

**Published:** 2020-06-12

**Authors:** Krzysztof Szyfter, Michał P. Witt

**Affiliations:** Institute of Human Genetics, Polish Academy of Sciences, ul. Strzeszyńska 32, 60-479 Poznań, Poland

**Keywords:** Amusia, Absolute pitch, Genes, Musicality

## Abstract

There is an agreement about joint genetic and environmental background of musical reception and performance. Musical abilities tend to cluster in families. The studies done on a random population, twins and families of gifted musicians provided a strong support for genetic contribution. Modern biomolecular techniques exploring linkage analysis, variation of gene copy number, scanning for whole-genome expression helped to identify genes, or chromosome regions associated with musical aptitude. Some studies were focused on rare ability to recognize tone without reference that is known as a perfect pitch where a far ethnic differentiation was established. On the other hand, gene deletion leading to dysfunction in amusical individuals also indicated appropriate loci “by negation.” The strongest support for an association of genes with musicality was provided for genes: *AVPR1* (12q14.2), *SLC6A4* (17q11.2), *GALM* (2p22), *PCDH7* (4p15.1), *GATA2* (3q21.3), and few others as well for 4q22, 4q23, and 8q13–21 chromosome bands.

## Introduction

Musicality attracts an attention of the general public as well as researchers because of its universal character. Defined as the capacity to perceive, reproduce, or create music, is recognized as a communication process involving pitch, rhythm, and timbre. As such, it is expected to share some brain functions and structures with speech. A suspected common root of speech and music perception indicated for the same brain attribution within right-hand auditory cortex, prefrontal cortex, and parietal areas of the brain (Stewart 2008, Peretz 2016, Brauchli et al. 2019). Further, an individual musicality spans from congenital amusia to absolute (perfect) pitch. Next, watching professional musicians, singers, and composers, raises the question why more talented persons are more frequent in some families and not in others. The latter question could evolve into consideration on hereditary (genetic) versus environmental background of musicality.

Studies on an impact of both potential contributing factors were postulated long ago (Shuter 1966). Such considerations should be also addressed to a link (or its lack) between perception and creation of music. The aim of the review is to describe findings concerning genetic background of musicality. Recently established molecular techniques, e.g., exploring DNA microarrays provided broad horizons that contributed vastly to results derived from classical genetic investigations.

There are no doubts concerning certain involvement of some physiological, cellular, and molecular processes activated by perceiving or creating music. It was a starting point to study individual differences in musicality explainable by genetic background (Stewart 2008, Peretz 2016, Gingras et al. 2015) assuming that music involvement should be detectable on the cellular and molecular level. It had been shown by demonstration of upregulation of microRNA fraction in peripheral blood as soon as 2 h after classical music performance. Five of the studied microRNA reached a level of significance: has-miR-3909, has-miR-30d-5p, has-miR-92a-3p, has-miR-222-3p, and has-miR-30a-5p (Nair et al. 2019). Recently genome-wide linkage and association scanning were employed to identify genes affecting auditory and creative involvement in music. Looking for gene copy number changes and gene expression estimation have become the main tools, and the results of these studies will be presented further on.

## Inheritance of musicality in families of famous musicians

A familial aggregation of musical abilities, like other artistic talents, is a well-known fact. The history of music contains many examples, the most spectacular of which is the family of Johann Sebastian Bach (1685–1750), one of the greatest musical geniuses of all time. The feature of transmitted musical abilities stretches there for over 200 years, for 6 generations of musicians (Table 1). Apart from Johann Sebastian himself, out of 35 musicians, at least six of his close relatives have, due to their particular artistic eminence, made a permanent mark in the history of music: in the generation preceding Johan Sebastian—the flutist Johann Ambrosius and organist Johann Christoph and two generations on, four sons, all outstanding composers—Wilhelm Friedemann, Carl Philip Emanuel, Johann Christian Friedrich, and Johann Christian (Wolff 1983). The fact is that the Bach family simply practiced the profession of a musician, which passed from father to son and thus earned a living. However, the presence in one family, in different generations as many as seven eminent musicians, one of whom is an undisputed genius, and at least two more very eminent, is difficult to describe as the result of family tradition only.

**Table 1:** Selected famous musicians and composers from Bach’s family

Person	Family link	Notices
Christoph	JS grandfather	1613–1661
		Composer, court musician
Johan Ambrosius	JS father	1645–1695
		Violinist, flutist
Johann Jacob	JS brother	1682–1722
		Oboist, composer,
Johann Ludwig	JS third cousin	1677–1731
		Composer, violinist
**Johann Sebastian**		1685–1750, composer, organist, music director
Wilhelm Friedemann	JS eldest son	1710–1781
		Composer, organist
Carl Phillipp Emanuel	JS second eldest son	1714–1788
		Composer, performer
Johann Christoph Friedrich	JS fifth son	1732–1795
		Composer, concert master
Johann Christian	JS youngest son,	1735–1782, composer,
	WF & CPE half-brother	Viola da gamba performer
Wilhelm Friedrich Ernst	JS grandson,	1759–1845
	JCF eldest son	Composer, music director

Among many Polish examples, the eminent instrumentalists of Wiłkomirski family comes to mind: violinist Michał, pianist Maria, cellist Kazimierz (for many years together performing as a family piano trio) and their stepsister violinist Wanda, an outstanding soloist of international stature and brother Józef - a conductor, long-time philharmonic director and organizer. They were all children of Alfred Wiłkomirski, a violinist, conductor ,and music teacher (Kydrynski 1960).

## Population, family association, and twin studies of musicality

Observations on familial aggregation of musicality stimulated development of appropriate research techniques. For making the studies on musicality testable and quantitatively measurable, the term musical aptitude was introduced as an ability to estimate rhythm, pitch, timbre, tone duration, and music structure. One of the measuring tools invented is Karma music test (KMT) applying small abstract sound patterns that are repeated to form hierarchic structures (Karma 1994). The normal (Gaussian) distribution of musical aptitude was found in population which supports conclusions from observations of family clusters of talented musicians. The conclusion on a significance of genetic factors in development of personal musicality (Oikkonen and Järvelä 2014) was further strongly supported by twin studies. The participants, monozygotic and dizygotic twin pairs of Caucasian origin were asked to distinguish incorrect sound in simple popular melodies (Drayna et al. 2001). The study was carried in terms of the distorted tunes test based on notes recognition with an incorrect pitch in simple popular melodies. Heritability was estimated for 71% (all data) and 80% after introducing certain cutoff value. The authors concluded that variation in musical pitch recognition is primarily due to highly heritable differences in auditory functions. Another study performed on Dutch twin pairs was addressed to such various domains of skill as music, arts, language, chess, mathematics, and sports. It was shown that shared environments have a small influence on skills including musical aptitude and talent and that genetic factors contribute significantly to a variation of musicality (Vinkhuyzen et al. 2009).

The group of Irma Järvelä (University of Helsinki, Finland), one of the most successful investigators in the field, contributed the results of studies on perception of music tested on four objective ways including Karma test. The genome–wide scanning for loci linked to musical aptitude indicated significantly to 4q22 and 8q13-21 loci (Pulli et al. 2008). The latter finding fits very well to the results published by Park et al. (2012) performed on the Mongolian population chosen due to a genetic homogeneity, decreased environmental heterogeneity, and restricted geographic distribution. After testing pitch-producing accuracy genome–wide scanning established a linkage of 4q23 locus. Within this locus, SNP of *UGT8* was revealed to be highly associated with musical ability. *UGT8* is known to be highly expressed in the central nervous system. Linkage of 4q22 and 4q23 with musical aptitude established by two research groups working on subjects derived from very different ethnicity but using almost identical experimental procedure was not unexpected as sound sensitivity studied in songbirds, mammals, primates, and human indicates for the same evolutionary tracts and suggests a high conservatory character of the appropriate genetic machinery (Oikkonen et al. 2016).

It was of interest to learn if musical aptitude and musical creativity are associated with the same genes. In Järvelä’s laboratory a cohort of Finnish families with already tested musicality and/or amateur involvement in music performance was studied. Polymorphism in selected genes (arginine vasopressin receptor 1A (*AVPR1*, 12q14.2), serotonin transporter (*SLC6A4*, 17q11.2), catechol-O-methyltransferase (*COMT*), dopamine receptor D2 (*DRD2*), and tyrosine hydroxylase 1 (*TPPH1*), all known from neurobiological studies to be active in communicative and creative processes, were estimated. The highest association with creativity was found for *AVPR1* (RS1 and RS3 haplotypes)*.* In various aspects of musical creativity, an impact of the genetic component was strong and as heritability was calculated for 0.84. Heritability coefficients of its particular components were equal for 0.40 for composing, 0.46 for arranging, and 0.62 for improvising. An impact of *AVPR1* in musical aptitude was not excluded in this study (Ukkola et al. 2009).

A role of *AVPR1* in musical creativity was already described in relation to dance performance. The study group consisted of dancers and their parents, athletes, and confronted with a control of nondancers/nonathletes. RS1 and RS3 haplotypes of *AVPR1* were significantly more frequent in dancers as compared with athletes and non-dancers. A difference between dancers and athletes was hypothetically explained by a spiritual activity in dancing that is not present in athletic competition (Bachner-Melman et al. 2005). Also, an association of *SLC64A* with successful choral singing was claimed by other authors (Morley et al. 2012).

Polymorphisms of the same two genes were investigated in the group of American white choral singers. Interestingly, none of the studied gene variants has reached a significant level. The authors’ conclusion is that choir singing depends at least partly on factors other than musical ability (Morley et al. 2012). An association of *AVPR1* with musicality was further confirmed in the study on Brazilian primary school children aged 7–5 years. Genotyping of *AVPR1* was studied together with other genes related to social and cognitive traits, namely *SLC6A4*, *ITGB3*, *COMT, DRD2*, and *DRD4.* Again *AVPR1* was the only gene found linked to musical ability (Mariath et al. 2017) that is in a close agreement with the findings described by Finnish investigators (Ukkola et al. 2009).

In search for another gene, genome-wide copy number variation (CNV) was studied in multigenerational families where study participants were estimated by three music tests including the Karma test, altogether leading to a combined music test score (COMB). Information about creativity in music was collected from a self-reported questionnaire. CNVs were detected predominantly in DNA regions coding for genes connected with neurodevelopment, learning, and memory. In this group, two genes were segregating with musicality: glucose mutarotase gene (*GALM*, 2p22) where locus duplication covering *GALM* co-segregated with musical creativity and protocadherin-α-gene cluster (*PCDHA 1-9*, 5q31) involved in neural migration, differentiation, and synaptogenesis co-segregating with low music tests score (Ukkola-Vuotti et al. 2013). Further genome-wide scanning for copy number variation in Finnish families where a good perception of music was relatively common showed the strongest association at 3q21.3 and 4p14 bands. Close to the first band, there is located Gata binding protein 2 gene (*GATA2*) and the second band harbors protocadherin 7 gene (*PCDH7*). Both discovered genes are involved in inner ear development: *GATA2* regulates the development of cochlear hair cells, while *PCDH7* plays a role in cochlear and amygdaloid complexes (Oikkonen et al. 2015).

Another study was undertaken to find a signature of positive selection attributed to musical aptitude. In the group of Finnish, individuals divided for high and low musical scores, three empirical metrics were applied to identify positive selection regions in the human genome connected with musical aptitude. The results indicated for several gene locations including genes affecting inner ear development, auditory perception (e.g., *USH2A*), cognition, and memory as well as language development (e.g., *FOXP1* and *VLDL*). The latter group of genes suggests that music and language share a common and evolutionary background (Liu et al. 2016).

An analysis of copy number variation served to look at ethnic differences of musicality phenotypes and corresponding genes. Gene variant distribution of genes/loci involved in musicality was compared in Finnish, Rwanda, Swedish, Swiss, and mixed population. Accumulation of certain gene variants in the Finnish population encourages to postulate a founder effect in the isolated Finnish population (Kanduri et al. 2013).

The most recent review paper by Järvelä (2018) tabulated 23 genes as top candidates for musical aptitude and music performance. All abovementioned genes are included. The results turned attention to α-synuclein gene (*SNCA*, 4q22) located in the most significant linkage region of musical aptitude on chromosome 4. *SNCA* is overexpressed when listening and performing music. The author stressed the role of genes active in dopaminergic pathway.

Gradually incoming information on newly identified genes associated with musicality does not rule out effect of environmental factors. Joint effect of genetic background and environmental influence raised over half of century ago (Shuter 1966) is still under intensive studies. At least a process of early musical training and family tradition should be considered. The study done on pairs of twins has shown that only a quarter of musical accomplishment was connected with genetics. A gene-environment interaction understood as genetic potential followed by hard practice brings someone to skilled performance (Hambrick and Tucker-Drob 2015). Larger literature evidence will be provided in the section describing absolute pitch.

## Absolute pitch

Absolute pitch (AP), known also as perfect pitch, is defined as the ability to identify or reproduce a musical pitch without reference (Brauchli et al. 2019). This is a rare skill difficult to population estimation due to a lack of commonly accepted precise definition and variety of estimation techniques applied. Early estimation at 1:10,000 adults was revised to as high as 1:1500 (Gingras et al. 2015). AP was found in only 1–2% of musical students and professional musicians, so it is not a prerequisite for the development of a musical career (Jobling 2014) and is actually a question of whether or not it helps.

Two points are to be mentioned before going into genetics of AP. First, there are drastic differences concerning occurrence of AP in children and adults. Adaptation of sound listening is one of the priorities determining child development. It is believed that little children prefer mother singing than talking to them (Mithen 2009). Lau et al. (2017) compared pitch perception in small groups of 3-months-old infants, 7-months-old babies and adults. The experiment has shown that 3-months-old infants were fully functional to discriminate the missing fundamental melodies. To preserve such an ability in early musical training is essential particularly for future conservatory students since a significant association between AP and the age at which an individual first began playing music was proved (Gregersen et al. 1999).

The second point is connected with an ethnic variation of AP prevalence. Gregersen et al. (1999) compared an occurrence of AP in Asian (*n* = 36) and non-Asian (*n* = 50) conservatory students finding it as 49.3% and 18.1%, respectively. The finding was confirmed later by comparison of AP in American vs. Chinese conservatory students (Deutsch et al. 2006), Chinese and Korean against other musicians (Hove et al. 2010) and Japanese (30% AP) vs. Polish (7% AP) music students (Miyazaki et al. 2012). It was hypothesized that differences could be connected with speech development because of deep tonality in East-Asian languages with Chinese at first (Deutsch et al. 2006). A fundamental impact of tonal language experience on AP performance was also discussed in the study performed on 37 AP possessors of mixed ethnicity (Van Hedger and Nusbaum 2019).

Studies on large samples of musicians showed that almost all displaying AP started their musical education by the age of 7. It was also suggested that it is rather unlikely that an individual is able to develop AP if musical training has been started after the age of 11 (Sergeant 1969). This notion does not hold exactly in the face of the most recent data since Van Hedger et al. (2019) showed that AP can be learned by some adults and the learned AP is undistinguishable from an inborn ability.

However, morphometric studies of brain structures have drawn attention to another biological agent that may affect the development of AP. It was found that AP musicians display a leftward asymmetry of a part of the temporal lobe called *planum temporale* (PT), a region historically associated with language and auditory processing. Although an early exposure to music may be a prerequisite for acquiring AP, the increased PT asymmetry in AP musicians may be determined in utero, strengthening a possibility of genetic background of PT asymmetry and thus of AP. This might create a functional dominance of the left *planum temporale* over the right one, which can provide an anatomical basis to facilitate the acquisition of AP. This structural difference is most likely caused by factors different from early musical training and can operate in such a way that young children with an inborn increased leftward PT asymmetry might develop AP if they have an early music exposure (Keenan et al. 2001).

AP characteristics was studied deeper by Athos et al. (2009) where piano tone pitch was investigated in participants of whom 44% were having extraordinary pitch-naming ability. The findings of this study were as follows: (i) pitch ability is not distributed randomly but is having bimodal distribution; the cluster of the best tone score suggests its genetic basis; (ii) AP tends to diminish with age that was observed in participants age span from 8 to 70 years.

The study of Smith et al. (2017) was not aiming into AP exclusively but its topic was pitch discrimination performance assessed in individuals of multiethnic ancestry. Better pitch performance was associated with higher intelligence, East Asian ancestry, male sex, younger age, and formal musical training (especially before age 6). A small sample size was analyzed by GWAS and gene-based collapsing analysis. No significant associations were identified even though chromosome regions (4q22, 4q23-4q26, chromosome 3), already shown as connected with pitch recognition (Smith et al. 2017).

Identification for genetic character of AP emerged from early observations on familial aggregation of AP (Profita and Bidder 1988, Baharloo et al. 1998). Nevertheless, a large study of Baharloo et al. (1998) performed on a group of professional musicians has shown that both early musical training and genetic predisposition are needed for the development of AP. Shortly, cooperative integration of genetics and environment makes AP to emerge. To determine an impact of genetic factor (Theusch and Gitschier (2011) performed AP segregation analysis in monozygotic twins (concordance equal to 78.6%), dizygotic twins (concordance 45.2%) and in their families. The conclusion was that AP is likely genetically heterogenous and is not inherited in a simple Mendelian fashion. Environmental, epigenetic, and stochastic factors also contribute to AP manifestation. The conclusion is consistent with the former publication of the research group. Nonparametric multipoint linkage analysis indicated significant linkage to 8q24.21 band in families of European ancestry. The same analysis established AP linkage with 7q22.3 band in families with East-Asian ancestry. Two more loci, namely 8q21.11 and 9p21.3 were linked to AP below statistical significance. The authors concluded that AP inheritance is genetically heterogeneous (Theusch et al. 2009).

The above finding has a strong support from studies on alteration of human transcriptome induced by listening to classical music. A presumption was that music listening alters molecular brain structure and function that was already shown by neurobiologic studies. Genome-wide transcription profile in peripheral blood lymphocytes of volunteers listening to Mozart Violin Concerto No 3 in G major for 20 min. The study group was divided further on music experienced and inexperienced participants. Differential gene expression (up- or downregulation) of 45 and 97 genes, respectively, was established in both groups of listeners. Upregulated genes are known to be involved in the secretion and transport of dopamine, neuron projection protein sumoylation, long-term potentiation, and dephosphorylation. One of the most upregulated genes was already mentioned *SNCA* (4q22.1) regulated by *GATA2* known to be associated with musical aptitude. In the group of upregulated genes, two genes linked to AP were identified, namely *FAM49B* (8q24.21) and *HDAC4* (2q37.3) (Kanduri et al. 2015a). By analogy Kanduri et al. (2015b) compared transcriptome of professional musicians after 2-h concert performance with music-free controls. The group of upregulated genes was similar to that found for listeners and included, among others, *SNCA* (4q22.1), *FOS* (14q24.3), and *DUSP1* (5q31.1) genes.

## Inborn defects (congenital amusia)

Significance of the genetic factor of musicality could be recognized also from analysis of disorders such as congenital amusia (CA), affecting approximately 1.5–4% of the population (Peretz and Vuvan, 2017). The other names in use are note deafness, tone or tune deafness, or dysmelodia. In extreme CA cases, affected take music as a type of a din. It was noted that amusia seems to be nonsyndromic which means it appears as the exclusive systemic deficiency. Definitely it is not associated with speech and prosody understanding. Further, an explanation by hearing deficiency, brain damage, and intellectual deficit was also excluded. Therefore, it was deduced that CA is connected with pitch processing that in turn is linked to structural defects in temporal and frontal cortices (Peretz 2008, Stewart 2008). Nevertheless, an association between musical auditory discrimination and intelligence remains an open question. Some studies excluded such an association but a large study done on Swedish twins showed a moderate correlation between IQ and musical auditory discrimination (Mosing et al. 2014).

An indication for genetic component of amusia was derived from the study of Peretz et al. (2007). The investigators were looking for congenital amusia in members of large families of amusic probands. Amusia was found in 39% of first-degree relatives in amusic families and only in 3% of control random families. Another study of the same research group done on a large cohort of Canadian subjects established prevalence of congenital amusia in 46% of first-degree relatives of probands. Other such associated disorders as dyslexia, speech disorder, memory problem, or spatial orientation difficulty were found only in a small minority of amusic participants. The authors concluded that congenital amusia may be influenced by several interacting genes (Peretz and Vuvan 2017). It is suggested that deregulations of the above-discussed genes such as *AVPR1A* (12q), *SLC6A4* (17q), and the loci identified on 8q and chromosome 4 would be responsible for triggering the disorder (Tan et al. 2014).

Similarly, Gingras et al. (2015) focused on such extremes of musical abilities as congenital amusia and absolute pitch. The authors raised the question of some rare monogenic effects still to be discovered; however, the most likely explanation of variability of musical perception and production is an involvement of variants of multiple interacting genetic loci (Gingras et al. 2015). Particular genes pointed to amusia are *FOXP*2 (7q31.2) (Gingras et al. 2015) and locus 22q11 (Gao et al. 2018). Mutations of the former in humans cause a severe speech and language impairments like developmental verbal dyspraxia (Lai et al. 2001). In singing birds, its ortholog is crucial for song learning and adult song performance (Adam et al. 2016), while in bats it conditions echolocation (Li et al. 2007).

## Association of music sensitivity with other symptoms

An interesting and useful field for investigation of music ability is a bilateral interaction of two cognitive tracts. The case is synesthesia, a neurological condition in which stimuli in one sensory realm (e.g., sound hearing) stimulate a perceptual experience in another realm (e.g. color). It means that an individual sees words, numbers, or musical notes as colors. Stimulation of colors by sounds is known as chromesthesia and it affects 0.05–1% of the population (Asher et al. 2009). The famous composers being holders of color synesthesia were: Franz Liszt, Olivier Messiaen, Alexander Scriabin, and Jean Sibelius, to name a few (Table 2).

**Table 2:** A list of genes and gene loci associated with musicality

Chromosome region	Gene	Claimed/proven function
4q22	*SNCA2*	Upregulation: musical aptitude, music listening, downregulation: amusia
8q13–21		Musical aptitude
3q21.3	*GATA2*	Music listening and performance
4q23 .	*UGT8*	Musical aptitude and musical creativity
12q14.2	*AVPR1*	Upregulation: musical aptitude and musical creativity, downregulation: amusia
5q31	*PCDHA 1–9*	Musical creativity
8q24.21		AP in families of European ancestry
7q22.3		AP in families with east-Asian ancestry
7q31.2	*FOXP*2	Amusia
22q11		Amusia
2q24		Auditory-visual synesthesia:

In search for genetic background of synesthesia, an analysis of whole-genome scanning and fine-mapping linkage study was performed. The following chromosome bands were indicated as associated with auditory-visual synesthesia: 2q24 (significant linkage), 5q33, 6p12, and 12p12 (suggestive linkages). Altogether the findings demonstrate an oligogenic character of the disorder (Asher et al. 2009). Tomson et al. (2011) added 16q12.2-23.1 as another suggestive linkage. The most recent publication (Tilot et al. 2018) applying whole-exome sequencing uncovered 37 genes co-segregating with synesthesia with special attention on *COL4A1*, *ITGA2*, *MYO10*, *ROBO3*, *SLC9A6*, and *SLIT2*. All these genes are expressed during early childhood when synesthetic associations are formed. Of note, there is an overlap of genes associated with absolute pitch and synesthesia. Linkage analysis has shown the LOD score peaks on chromosomes 6 and 2 (Gregersen et al. 2013). Japanese investigators showed that synesthesia can also emerge in non-AP individuals (Itoh and Nakada 2018).

Another disease studied in connection with musicality is Williams syndrome (WS): a multisystem disorder characterized by dysmorphic facial features, moderate intellectual disability, connective tissue abnormalities, and unique cognitive profile. Genetic studies have shown a large 1.8 Mb deletion of 26 contiguous genes of chromosome 7q11.23. The deleted genes include *NCF1*, *SVAS*, elastin (*ELN)* and *MAG12.* The latter gene is associated with severe cognitive disability that could explain anxiety to noise including music (Morris 2010). The specific phenotype of children with WS includes a particular interest in music and despite of average IQ 50–60 many possess special cognitive skills, both verbal and musical with an outstanding memory for songs, sense of rhythm, and remarkable auditory acuity in parallel to their speech and language aptitude exceeding their other cognitive functions (Bellugi et al. 1999). Some WS individuals exhibit perfect pitch (Lenhoff et al. 2001).

## Conclusions

It has to be noted that music, being one of “high art” vital components, is also a part of human biology and, as such, had a significant impact on human evolution and development: it had an adaptive influence causing an increase of reproductive chances and intensification of human bonds, both familial, and social. With music, humans evolved as musical species, since music was just one of the essential elements shaping the developing human brain. Vocalization, as an early manifestation of creative musicality, occurs in *Hominidae*, and in some it has been analyzed and finely translated (gibbons, chimpanzees). Proto-musical language, probably used by *Homo sapiens* at the time of his speciation, grew out of these vocalizations some 200,000 years ago, becoming the basis of non-verbal communication. Music, as a biological phenomenon, had to be shaped by genes what is best exemplified by extended pedigrees of musical families like the Bachs and certain disorders involving musicality. A multitude of so far collected data shows that music might be even a better denominator of genetic distances than language, i.e., high musical similarity predicts high genetic similarity (Pamjav et al. 2012). Thus, together with the recent progress in compound phenotype mapping of multigenic/multifactorial traits like musical aptitude (Gingras et al. 2015) could entail an even deeper exploration of the relationship between music and genetics.

## References

[CR1] Asher JE, Lamb JA, Brocklebank D et al. (2009) A whole genome scan and fine-mapping linkage study of auditory-visual synesthesia reveals evidence of linkage to chromosome 2q24, 5q33, 6p12, and 12p12. Am J human genet 84,279:285. doi: 10.1016/j.ajhg.2009.01.012 10.1016/j.ajhg.2009.01.012 PMC2668015 19200526

[CR2] Adam I, Mendoza E, Kobalz U, Wohlgemuth S, Scharff C (2016) FoxP2 directly regulates the reelin receptor VLDLR developmentally and by singing. Molecular and Cellular Neuroscience 74:96–105 10.1016/j.mcn.2016.04.002 27105823

[CR3] Athos E, Levinson B, Kistler A, Zemansky J, Bostrom A, Freimer N, Gitschier J (2009) Dichotomy and perceptual distortions in absolute pitch ability. Proc Natl Acad Sci U S A 104:14795–14800. 10.1073/pnas.0703868104 10.1073/pnas.0703868104 PMC1959403 17724340

[CR4] Bachner-Melman R, Dina C, Zohar AH, Constantini N, Lerer E, Hoch S, Sella S, Nemanov L, Gritsenko I, Lichtenberg P, Granot R, Ebstein RP (2005). AVPR1a and SLC6A4 gene polymorphisms are associated with creative dance performance. PLoS One.

[CR5] Baharloo S, Johnston PA, Service SK, Gitschier J, Freimer NB (1998). Absolute pitch: an approach for identification of genetic and nongenetic components. Am J Human Genet.

[CR6] Brauchli C, Leipold S, Jäncke L (2019). Univariate and multivariate analyses of functional networks in absolute pitch. Neuroimage.

[CR7] Deutsch D, Henthorn T, Marvin E, Xu H (2006) Absolute pitch among American and Chinese conservatory studentsd: prevalence differences, and evidence for a speech-related critical period. J Acoust Soc am 119719-22. doi: 10.1121/1.2151799 10.1121/1.2151799 16521731

[CR8] Drayna D, Manichaikul A, de Lange M, Snieder H, Spector T (2001). Genetic correlates of musical pitch recognition in humans. Science.

[CR9] Gao L, Tang SX, Yi JJ (2018). Musical auditory processing, cognition, and psychopathology in 22q11.2 deletion syndrome. Am J Med Genet B Neuropsychiatr Genet.

[CR10] Gingras B, Honing H, Peretz I, Trainor LJ, Fisher SE (2015). Defining the biological bases of individual differences in musicality. Philosoph Transact B.

[CR11] Gregersen PK, Kowalsky E, Kohn N, Marvin EW (1999). Absolute pitch: prevalence, ethnic variation, and estimation of the genetic component. Am J Human Genet.

[CR12] Gregersen PK, Kowalsky E, Lee A, Baron-Cohen S, Fisher SR, Asher JE, Ballard D, Freudenberg J, Li W (2013). Absolute pitch exhibits phemitypic and genetics overlap with synthesia. Human Molec Genet.

[CR13] Hambrick DZ, Tucker-Drob EM (2015). The genetics of music accomplishment: evidence for gene-environment correlation and interaction. Psychon Bull Rev.

[CR14] Hove MJ, Sutherland ME, Krumhansi CL (2010). Ethnicity effects in relative pitch. Psychon Bull Rev.

[CR15] Itoh K, Nakada T (2018). Absolute pitch is not necessary to pith class-color synesthesia. Consious Cogn.

[CR16] Järvelä I (2018). Genomics studies on musical aptitude, music perception, and practice. Ann N Y Acad Sci.

[CR17] Jobling MA (2014) The music of the genes. Investigative Genetics 5 (1):2 10.1186/2041-2223-5-2 PMC3901330 24461062

[CR18] Kanduri C, Kuusi T, Ahvenainen M, Philips AK, Lähdesmäki H, Irma Järvelä I (2015). The effect of music performance on thetranscriptome of professional musicians. Sci Rep.

[CR19] Kanduri C, Raijas P, Ahvenainen M, Philips A, Ukkola-Vuoti L, Lähdesmäki H, Järvelä I (2015). The effect of listening to music on human transcriptome. Peer J.

[CR20] Kanduri Ch, Ukkola-Vuoti L. Oikkonen J et al. (2013) The genome-wide landscape of copy number variations in the MUSGEN study provides evidence for a founder effect in the isolated Finnish population. Eur J Human Genet 21:1411–1416. doi: 10.1038/ejhg.2013.60 10.1038/ejhg.2013.60 PMC3831076 23591402

[CR21] Karma K (1994). Auditory and visual temporal structuring: how important is sound to musical thinking. Psychol Music.

[CR22] Keenan JP, Thangaraj V, Halpern AR (2001). Sclaug G. Absolute pitch and planum temporale NeuroImage.

[CR23] Kydryński L (1960). Wanda Wiłkomirska.

[CR24] Lau BK, Lalonde K, Oster MM, Werner LA (2017). Infant pitch perception: missing fundamental melody discrimination. J Acoust Soc Am.

[CR25] Lenhoff HM, Perales O, Hickok G (2001) Absolute Pitch in Williams Syndrome. Music Perception 18 (4):491–503

[CR26] Li G, Wang J, Rossiter SJ, Jones G, Cotton JA, Zhang S (2008) The hearing gene Prestin reunites echolocating bats. Proceedings of the National Academy of Sciences 105 (37):13959–13964 10.1073/pnas.0802097105 PMC2544561 18776049

[CR27] Liu X, Kanduri C, Oikkonen J (2016). Detecting signatures of positive selection associated with musical aptitude in the human genome. Sci Rep.

[CR28] Mariath LM, Silva AM, Kowalski TW, Gattino GS, Araujo GA, Figueiredo FG, Tagliani-Ribeiro A, Roman T, Vianna FSL, Schuler-Faccini L, Schuch JB (2017). Music genetics research: association with musicality of a polymorphism in the AVPR1A gene. Genet Molec Biol.

[CR29] Mithen S (2009). The music instinct. The evolutionary basis of music. Ann N Y Acad Sci.

[CR30] Miyazaki K, Makomaska S, Rakowski A (2012). Prevalence of absolute pitch: a comparison between Japanese and Polish music students. J Acoust Soc Am.

[CR31] Morley AP, Narayanan M, Mines R, Molokhia A, Baxter S, Craig G (2012). AVPR1A and SLC6A4 polymorphisms in choral singer and non-musicians: a gene association study. PLoS One.

[CR32] Morris CA (2010). Introduction: Williams syndrome. Am J Med Genet C Semin Med Genet.

[CR33] Mosing MA, Pedersen NL, Madison G, Ullén F (2014). Genetic pleiotropy explains associations between musical auditory discrimination and intelligence. PLoS One.

[CR34] Nair PS, Kuusi T, Ahvenainen M, Phillips AK, Järvelä I (2019). Music performance regulates microRNAs in professional musicians. Peer J.

[CR35] Oikkonen J, Huang Y, Onkano P (2015). A genome-wide linkage and association study of musical aptitude identifies loci containing genes related to inner ear development and neurocognitive functions. Mol Psychiatry.

[CR36] Oikkonen J, Järvelä I (2014). Genomics approaches to study musical aptitude. Bioessays.

[CR37] Oikkonen J, Onkamo P, Järvelä I, Kanduri C (2016) Convergent evidence for the molecular basis of musical traits. Sci Rep 6:e39707. 10.1111/nyas.13620 10.1038/srep39707 PMC5177873 28004803

[CR38] Pamjav H, Juhász Z, Zalán A, Németh E, Damdin B (2012) A comparative phylogenetic study of genetics and folk music. Molecular Genetics and Genomics 287(4):337–349 10.1007/s00438-012-0683-y 22392540

[CR39] Park H, Lee S, Kim H-J, Ju YS, Shin J-Y, Hong D, von Grotthuss M, Lee D-S, Park C, Kim JH, Kim B, Yoo YJ, Cho S-I, Sung J, Lee C, Kim J-I, Seo JS (2012). Comprehensive genome analyses associate UGT8 variants with musical ability in a Mongolian population. J Med Genet.

[CR40] Peretz I (2008) Musical disorders. From behavior to genes. Curr direct Psychol Sci. 17:329-333. 10.1111/j.1467-8721.2008.00600.x

[CR41] Peretz I (2016). Neurobiology of congenital amusia. Trends Cognit Sci.

[CR42] Peretz I, Cummings S, DubéMP (2007) The genetics of congenital amusia (tone deafness): a family-aggregation study. Am J Human Genet 821:582–588. doi: 10.1086/521337 10.1086/521337 PMC1950825 17701903

[CR43] Peretz I, Vuvan DT (2017). Prevalence of congenital amusia. Eur J Human Genet.

[CR44] Profita J, Bidder TG (1988). Perfect pitch. Am J Med Genet.

[CR45] Pulli K, Karma K, Norio R, Sistonen P, Göring HHH, Järvelä I (2008). Genome-wide linkage scan for loci of musical aptitude in Finnish families: evidence for a major locus at 4q22. J Med Genetics.

[CR46] Sergeant D (1969) Experimental Investigation of Absolute Pitch. Journal of Research in Music Education 17 (1):135

[CR47] Shuter R (1966). Hereditary and environmental factors in musical ability. Eugenics Rev.

[CR48] Smith LM, Bartholomew AJ, Burnham LE, Tillman B, Cirulli ET (2017). Factors affecting pitch discrimination performance in a cohort of extensively phenotyped healthy volunteers. Sci Rep.

[CR49] Stewart L (2008). Fractionating the musical mind: insights from congenital amusia. Curr Opin Neurobiol.

[CR50] Tan YT, McPherson GE, Peretz I, Berkovic SF, Wilson SJ (2014) The genetic basis of music ability front Psychol 5:art 658. doi: 10.3389/fpsyg.2014.00658 10.3389/fpsyg.2014.00658 PMC4073543 25018744

[CR51] Teusch E, Basu A, Gitschier J (2009). Genome-wide study of families with absolute pitch reveals linkage to 8q24.21 and locus heterogeneity. Am J Human Genet.

[CR52] Teusch E, Gitschier J (2011). Absolute pitch twin study and segregation analysis. Twin Res Human Genet.

[CR53] Tilot AK, Kucera KS. Vino A, Asher JE, Baron-Cohen S, Fisher SE (2018) Rare variants in axonmogenesis genes connect three families with sound-color synsthesia. PNAS 115:3168–3173. doi: 10.1073/pnas.1715492115 10.1073/pnas.1715492115 PMC5866556 29507195

[CR54] Tomson SN, Avidan N, Lee K, Sarma AK, Tushe R, Milewicz DM, Bray M, Leal SM, Eagleman DM (2011). The genetics of coloed sequence synsthesis: suggestive evidence of linkage to 16q and genetic heterogeneity for the condition. Behav Brain Res.

[CR55] Ukkola LT, Onkamo P, Raijas P, Karma K, Järvelä I (2009). Musical aptitude is associated with AVPR1A-haplotypes. PLoS One.

[CR56] Ukkola-Vuoti L, Kanduri C, Oikknonen J (2013). Genome-wide copy number variation analysis in extended families and unrelated individuals characterized for musical aptitude and creativity in music. PLoS One.

[CR57] Van Hedger SC, Heald SLM, Nusbaum HC (2019). Absolute pitch can be learned by some adults. PLoS One.

[CR58] Van Hedger SC, Nusbaum HC (2019). Individual differences in absolute pitch performance: contributions of working memory, musical expertise and tonal language background. Acta Psychol.

[CR59] Vinkhuyzen AAE, van der Sluis S, Posthuma D, Boomsma DI (2009) The heritability of aptitude and exceptional talent across different domains in adolescents and young adults. Behavior Genetics 39 (4):380–392 10.1007/s10519-009-9260-5 PMC2688647 19288254

[CR60] Wolff C (1983). The new grove Bach family.

